# Immunoregulatory role of platelet derivatives in the macrophage-mediated immune response

**DOI:** 10.3389/fimmu.2024.1399130

**Published:** 2024-06-25

**Authors:** Eduardo Anitua, María Troya, Mohammad H. Alkhraisat

**Affiliations:** ^1^ Regenerative Medicine Laboratory, BTI-Biotechnology Institute, Vitoria, Spain; ^2^ University Institute for Regenerative Medicine & Oral Implantology, UIRMI (UPV/EHU-Fundación Eduardo Anitua), Vitoria, Spain

**Keywords:** macrophages, polarization, platelet rich plasma, platelet lysate, platelet rich fibrin

## Abstract

**Background:**

Macrophages are innate immune cells that display remarkable phenotypic heterogeneity and functional plasticity. Due to their involvement in the pathogenesis of several human conditions, macrophages are considered to be an attractive therapeutic target. In line with this, platelet derivatives have been successfully applied in many medical fields and as active participants in innate immunity, cooperation between platelets and macrophages is essential. In this context, the aim of this review is to compile the current evidence regarding the effects of platelet derivatives on the phenotype and functions of macrophages to identify the advantages and shortcomings for feasible future clinical applications.

**Methods:**

A total of 669 articles were identified during the systematic literature search performed in PubMed and Web of Science databases.

**Results:**

A total of 27 articles met the inclusion criteria. Based on published findings, platelet derivatives may play an important role in inducing a dynamic M1/M2 balance and promoting a timely M1-M2 shift. However, the differences in procedures regarding platelet derivatives and macrophages polarization and the occasional lack of information, makes reproducibility and comparison of results extremely challenging. Furthermore, understanding the differences between human macrophages and those derived from animal models, and taking into account the peculiarities of tissue resident macrophages and their ontogeny seem essential for the design of new therapeutic strategies.

**Conclusion:**

Research on the combination of macrophages and platelet derivatives provides relevant information on the function and mechanisms of the immune response.

## Graphical Abstract

Created with BioRender.com. TRM, tissue-resident macrophages.

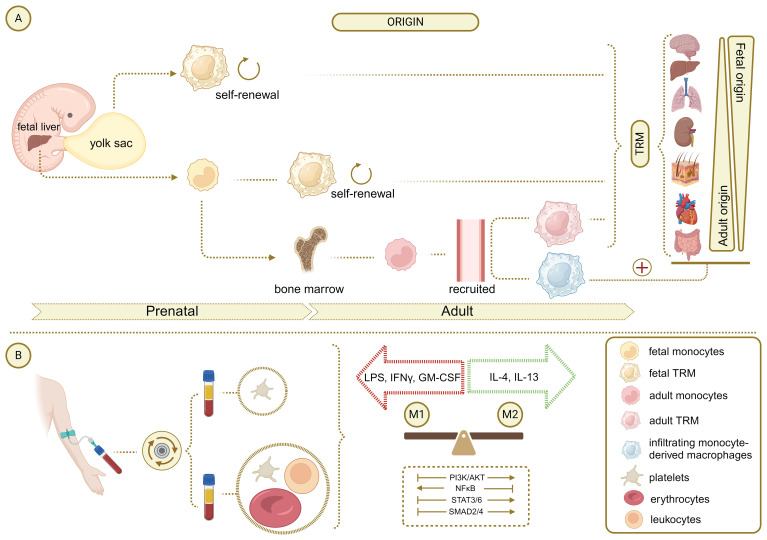

## Introduction

Macrophages are innate immune cells present in all tissues. Beyond their central role in innate immunity, they are also crucial for organ development, inflammatory response, tissue remodelling and homeostasis (1, 2). They display a remarkably phenotypic heterogeneity and functional plasticity as they are epigenetically programmed in response to different microenvironmental cues (3–5).

Macrophages were first discovered by Elie Metchnikoff in the late 19th century (6). For decades, the concept of mononuclear phagocyte system (MPS), proposed by van Furth et al. (7, 8), has prevailed. This theory held the idea that tissue macrophages derived entirely from adult blood monocytes originating from bone marrow progenitors (9–11). However, accumulating evidence from fate-mapping mouse models and parabiosis studies have revised this paradigm regarding cellular ontogeny (12, 13). These data establish that most tissue-resident macrophages (TRM) arise from embryonic precursors and persist into adulthood due to its self-renewal ability and independently of adult hematopoietic stem cells (12, 14). Embryonic- and adult- derived macrophages coexist in certain tissues, and the contribution of each particular subset depends on the type of tissue (1, 11, 12) thus creating a complex scenario. Some tissues, such as dermis, gut and heart require a continuous blood monocyte replenishment during adulthood (10, 13, 15). Conversely, the microglia self-renewal is independent of adult haematopoiesis, deriving almost exclusively from embryonic progenitors (16, 17).

Macrophage polarization refers to a rigorously controlled process by which macrophages display different functional phenotypes in response to microenvironmental stimuli (18, 19). Overall, it is considered that macrophages can polarize into classically activated macrophages (M1) and alternatively activated macrophages (M2) (2, 20). This concept denotes an oversimplification of the M1/M2 paradigm as this is rather represented by a dynamically continuum of activation states (21, 22). M1 macrophages are polarized by bacterial endotoxins such as lipopolysaccharide (LPS) and Th1-related cytokines including interferon gamma (IFN-γ) and granulocyte-macrophage colony-stimulating factor (GM-CSF). As a result, they produce pro-inflammatory cytokines. Therefore, they exhibit a pro-inflammatory phenotype with antimicrobial and antitumoral activities (18, 20, 23). Moreover, M1 macrophages are involved in matrix degradation by direct and indirect production of matrix metalloproteinases (MMPs) and a variety of antifibrotic cytokines such as CXCL10 (18, 24, 25). Conversely, M2 macrophages are activated by Th2 cytokines including IL-4 and IL-13 and express high level of scavenger proteins such as mannose receptor (CD206) (18, 26, 27). M2 macrophages can be further subdivided *in vitro* into M2a, M2b, M2c and M2d, in accordance with their activation stimuli and gene expression profile (22, 28, 29). Functionally, M2 macrophages are associated with the resolution of inflammation and the promotion of angiogenesis and tissue repair (19, 30). Both phenotypes also differ in their metabolic profiles. The metabolism of M1 macrophages is rely on glycolysis, whereas M2 macrophages obtain their energy through fatty acid oxidation (19, 31). Macrophages are essential for maintaining tissue homeostasis, thus an imbalance between both phenotypes is present in many diseases. Due to their involvement in the pathogenesis of several human conditions, macrophages are considered as an attractive therapeutic target (32–34).

Regarding the use of platelets derivatives such as platelet-rich plasma (PRP), platelet lysate (PL) or platelet-rich fibrin (PRF), they have been successfully applied in many medical fields including dentistry, orthopedics, sports medicine, ophthalmology, dermatology and gynecology (35–40). The widespread clinical use of these platelet derivatives relies on their capacity to feature in different processes, beyond tissue hemostasis. They also exert their role in many biological processes, such as inflammation, immunity, angiogenesis and tissue regeneration (36, 41–43). Platelet derivatives provide growth factors, cytokines, chemokines and other biological mediators. They also contain clotting factors for developing a fibrin-based scaffold (36, 44). As active participants in innate immunity, cooperation between platelets and macrophages is essential (45). Platelets promote their recruitment and activation (46) and are involved in the macrophage NLRP3 inflammasome activation (47). Platelets express many immunomodulatory molecules such as adhesion receptors (P-selectin), or multiple pattern-recognition receptors including members of the Toll-like receptors (TLR) family (45, 48, 49). Moreover, they release a plethora of biological mediators including a large amount of chemokines (e.g., CXCL4) that may modulate the responses of macrophages (41, 42, 50). Nevertheless, the complexity of this partnership means that its mechanisms are not yet fully understood. Moreover, the lack of standardization for these platelets products leads to multiple preparation protocols with different concentration, composition or activation state resulting in biologically heterogeneous final products (51, 52) that may have an impact on the final response hindering the evaluation of clinical effectiveness.

As in regenerative medicine successful treatments usually emerge from the synergy of combining different treatments, the aim of this review is to gather the current evidence on the effects of platelet derivatives on the phenotype and functions of macrophages to identify the advantages and shortcomings for feasible future clinical applications.

## Material and methods

### Literature search

For this narrative review, a systematic literature search was performed in PubMed and Web of Science database in August 2023, using the following search strategy: “(((((((platelet rich plasma) OR (PRP)) OR (platelet lysate)) OR (PL)) OR (platelet rich fibrin))) OR (PRF)) AND (macrophage polarization)”. Papers were excluded if: 1) the article was written in any language other than English or Spanish 2) duplicates 3) reviews, perspectives, editorials, commentaries, thesis, book chapters 4) no full-text available 5) out of scope 6) did not include macrophages 7) did not include PRP, PRF, PL or similar.

### Data extraction

Studies that passed the initial title and abstract evaluation were retrieved for full-text review. For data extraction, an evidence table was created with Microsoft Excel. The following data were included: author and year of publication, study type, field of application, macrophages’ origin, platelet derivatives’ issues (type, origin, sample size, type of anticoagulant, conditions of centrifugation, presence or absence of leukocytes, type of activator), comparison groups, macrophage polarization and summary results.

### Assessment of reporting quality and risk of bias

The reporting quality and the risk of bias were assessed according to the criteria reported by Golbach et al. (53). The reporting quality was determined by the presence (“yes/partly”) or absence (“no”) of critical information. According to the answers (yes”, “partly”, or “no”), the risk of bias was divided into 3 categories: low, moderate and high, respectively.

## Results

**Figure 1:**
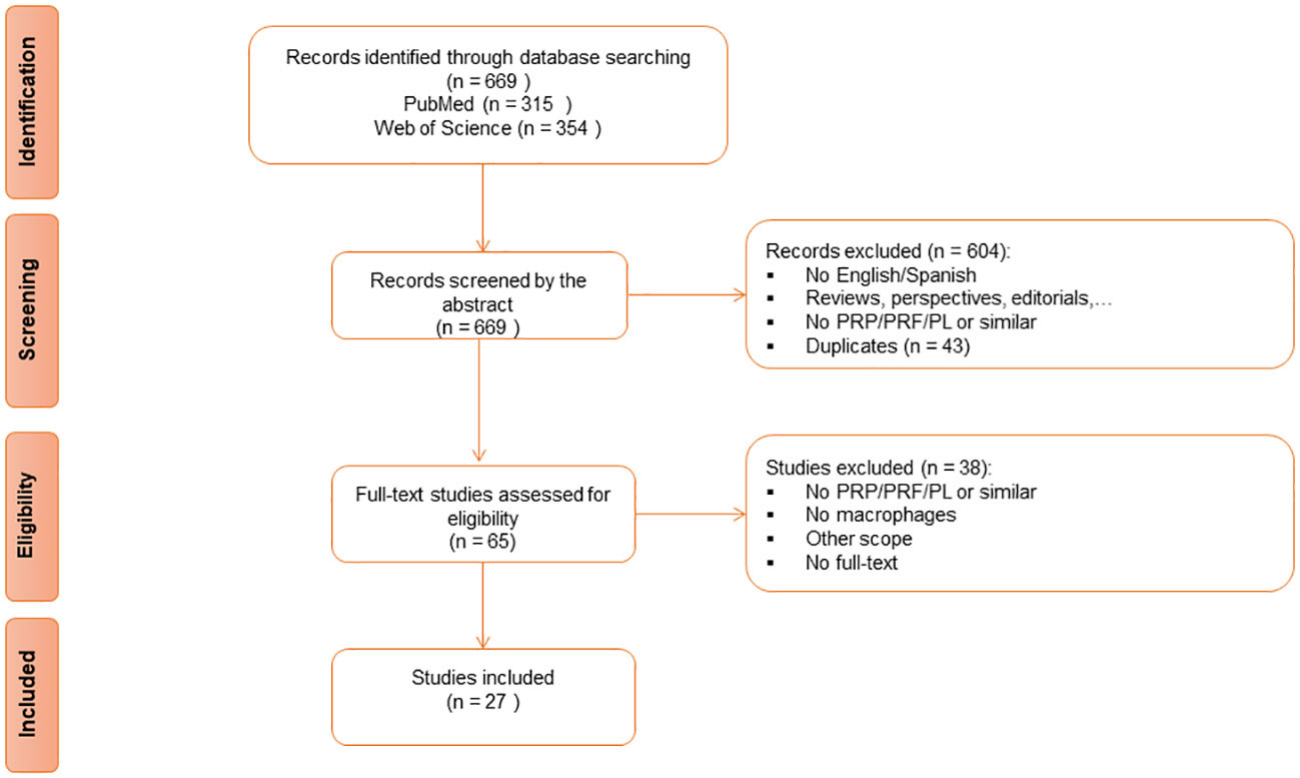
Preferred Reporting Items for Systematic reviews and Meta-Analyses (PRISMA) Flow Diagram for Study Selection.

The search strategy yielded a total of 669 articles from the two databases. Forty-three articles were removed as duplicates. After a properly screening, 27 studies (54–80) were finally included for the analysis in this review (**Figure 1**, **Table 1**).

**Table 1:** Summary information of the studies included in this review.

Ref.	Study type	Field of application	Macrophages’ origin	Comparison groups	Polarization	Summary results
Cenni et al., 2010 (55)	*In vitro*	Bone regeneration	Osteoclasts derived from human PBMC	**Group I:** no supplements (negative control) **Group II**: RANKL and M-CSF **Group III**: PRP w/ or w/o suppl **Group IV:** PPP w/ or w/o suppl	Impairs osteoclast generation	The multinucleated cells incubated with PRP supernatant at 10% showed a significantly low bone resorptive activity, as it was demonstrated by the reduced ability to degrade collagen type I. PRP interfered with the complete differentiation process and osteoclast activation. At high dosage it affected osteoclast formation also at an early stage of differentiation.
He et al. 2021 (58)	*In vitro*	Bone regeneration	THP-1	**Group I:** 10%FBS **Group II:** 10%hPL **Group III**: 5%hPL (monoculture: THP-1 and coculture: JPCs and THP-1)	Inhibition of M1 polarization	Under 5% hPL conditions, the pseudopodia of M1 macrophages were shown to be longer than those of M2 macrophages, while the opposite was observed under 10% FBS supplementation. CD 86 expression of M1 macrophages cultured in 5% hPL was significantly higher compared to CD86 expression in M2 macrophages, whereas no significant differences between M1 and M2 macrophages were detected in 10% FBS- and 10% hPL-supplemented medium. Compared with FBS and 10% hPL, culture supplementation with 5% hPL was superior for the generation of distinct THP-1-derived M1 and M2 macrophage phenotypes. The numbers of M1/M2 macrophages can be decreased in coculture with JPCs under 5% hPL conditions compared to other supplementations. Under hPL culture condition, JPCs were best able to effectively inhibit M1 polarization of macrophages in the direct coculture system.
O’Donnell et al. 2019 (66)	*In vitro*	OA	Human PBMC	**Group I:** negative control (serum free complete medium) **Group II:** positive control (+LPS) **Group III**: H-PRP **Group IV**: OA-PRP	OA-PRP: towards M1	OA-PRP resulted in significant upregulation of mRNA for inflammatory proteins in human macrophages, which was not apparent after treatment with PRP from healthy young males. PRP from older males with OA stimulated an inflammatory phenotype in human macrophages *in vitro*. These data suggested that age and OA disease state may influence the clinical response to PRP treatment.
Uchiyama et al. 2021 (74)	*In vitro*	OA	Human PBMC	**Group I**: MDM control **Group II**: M1 polarized **Group III:** M2 polarized **Group IV**: M1-M2 polarized **Group V:** LP-PRP-added **Group VI:** APS-added	Towards M2	LP-PRP and APS differed in the concentrations of cytokines involved in macrophage polarization with higher concentrations of both M1 and M2 macrophage related factors in APS. The addition of PRP supernatants decreased the expression of M1 macrophages markers, but there was no difference between the purification kits. The expression of M2 macrophage surface markers tended to be maintained or increased by the addition of PRP supernatants while the gene expression of IL-10 increased in the APS-added group and TGF-β increased in the LP-PRP-added group. PRPs could repolarize M1 macrophages to M2 macrophages. LP-PRP promoted the polarization to M2c macrophages and that APS specifically promoted the polarization to M2a macrophages.
Escobar et al., 2018 (56)	*In vitro*	Tissue regeneration	Human PBMC	**Group I:** P-PRP **Group II:** S-PRP **Group III:** Ca^2+^	Different profiles of tissue-repair macrophages	The expression levels of CD206 increased in a dose-dependent manner. Macrophages stimulated with either P-PRP or S-PRP presented no differences in CD206 expression, however, both CD163 and CD86 presented higher expression levels when macrophages were stimulated with S-PRP instead of P-PRP. The IL-10 production in P-PRP-stimulated macrophages was higher than S-PRP-stimulated macrophages, however, no differences were obtained in TNF-α production. Macrophages stimulated with P-PRP produced tenfold higher levels of IL-10 when compared with unstimulated macrophages. Furthermore, IL-10/TNF-α production ratio was sevenfold higher in P-PRP-stimulated macrophages than S-PRP-stimulated macrophages.
GraÇa et al. 2022 (57)	*In vitro*	Tissue regeneration	THP-1	**Group I:** unstimulated cells (control) **Group II:** sEVs **Group III**: mEVs	sEVs: to a hybrid M1/M2 phenotype mEVs: to a non-polarized state.	The M1/M2 ratio was 1.0 ± 0.3, 5.8 ± 1.7 and 2.2 ± 1.1 when macrophages were cultured under unstimulated, sEVs and mEVs conditions, respectively. sEVs induced macrophage elongation more frequently than under unstimulated and mEVs conditions, which could be correlated with the polarization towards the M2 phenotype. sEVs significantly upregulated the levels of the M2-marker IL-15, compared to unstimulated macrophages. mEVs showed a similar expression of both M1- and M2-markers. sEVs augmented the release of pro-inflammatory cytokines interleukin (IL)-1β, IL-6, IL-8 and granulocyte-macrophage colony-stimulating factor (GM-CSF), and to a lesser extent, anti-inflammatory IL-10, when compared with unstimulated macrophages, which might be associated with their differentiation towards the hybrid M1/M2 phenotype. The release profile for the mEV group was similar to non-polarized macrophages.
Luo et al. 2021 (63)	*In vitro*	Tissue regeneration	THP-1	**Group I**: Control **Group II**: 10% CCM **Group III**: 20% CCM **Group IV:** 50% CCM	Towards M2	CGF promoted monocyte migration. CCM treatment promoted non-adherent THP-1 monocyte differentiation into adherent macrophages. The mRNA expression of the M2 marker CD163 was significantly upregulated with increasing CCM concentrations, but the M1 marker CD80 was downregulated upon CCM treatment. These data indicated that CGF had a functional effect on the immune response by suppressing the inflammatory cytokine secretion and enhancing chemokine production in immune cells. AKT pathway was activated in CGF-mediated signalling dynamics. CGF induced THP-1 monocyte/ macrophage transition, promoted macrophage polarization and modulated cytokine secretion *in vitro*, which correlated with the AKT pathway activation.
Papait et al. 2018 (67)	*In vitro*	Tissue regeneration	Human PBMC	**Group I:** iDC **Group II:** DC-ST **Group III:** DC-LD	Anti-inflammatory	Upon stimulation with LPS, DC-LD secreted more IL-10 and PGE2 than DC-ST. PRP inhibited the differentiation of monocytes to CD1a+ dendritic cells and favoured the expansion of phagocytic CD163+CD206+ fibrocyte-like cells that did not produce IFN-γ. PRP-ST and PRP-LD induced DC with similar features. They promoted the expansion of regulatory CD4+CD25+FoxP3+ T cells upon allostimulation or antigen specific priming. Allogeneic PRP could foster the differentiation of monocytes to a regulatory anti-inflammatory population possibly favouring wound healing.
Scopelliti et al. 2021 (71)	*In vitro*	Tissue regeneration	Human PBMC	**Group I**: ctr **Group II:** PL	Towards M2	PL reprogramed M (IFNγ+LPS) macrophages towards a M2‐like phenotype, by reducing the expression of CD80 and CD86 costimulatory molecules, decreasing the production of TNF‐α, of the chemokine CXCL10 and enhancing the release of TGF‐β and the expression of the M2‐marker CD206, CD200R, PPAR and arginase. NFκB expression was also significantly reduced. The release of TGF‐β and the signalling SMAD2 and SMAD4, in particular, were significantly increased. The supernatant of PL‐treated macrophages significantly promoted fibroblast expression of type‐I collagen and to a lesser extent of type-III collagen. Surprisingly, PL downregulated the release of IL-10, which, instead, was reported to be significantly upregulated by the M2 macrophages.
Tylek et al. 2019 (73)	*In vitro*	Tissue regeneration	Human PBMC	**Group I**: FCS **Group II:** hS **Group III:** hPL+ heparin **Group IV**: hPL- heparin	The macrophage phenotype was conserved in medium with hPL and resembled that of spontaneously differentiated M0. In co-culture rather an M1-like type	In M0 cell cultures with hPL, spontaneously differentiated macrophages showed differences in M2 marker expression to those cultivated in hS- and FCS- supplemented cultures. A decrease of CD163 and an increase of CD206 expression was observed for hPL-supplemented cultures. Macrophages cultured in medium supplemented with hPL reflected a more similar expression pattern to cells in hS- than in FCS-containing media. The expression profile of each cytokine varied for different sera supplementation. The release of IL-1β was downregulated in macrophages cultivated in medium with FCS, compared to those cultivated in medium with hS and hPL+/− heparin. The highest expression of IL-6 was observed in the hPL+ heparin group, whereas the lowest level was detectable in FCS. IL-8 was highly expressed in all tested culture conditions. The anti-inflammatory-related cytokine IL-10 was generally released at lower levels with the highest values for macrophages in media with hPL+ heparin. Only macrophages cultivated with hPL+ released significantly higher amounts of the cytokines IL-1β and IL-6 into the cell culture media. Heparin had a stimulating effect on the cytokine release of macrophages.
Yadav et al. 2022 (77)	*In vitro* and *in vivo*	Nerve Regeneration	THP-1	*In vitro:* **Group I**: M0 **Group II**: M1 **Group III**: M0+PRGF **Group IV**: M1+PRGF *In vivo*: **Group I**: Sham **Group II**: Saline **Group III**: PRGF	Towards M2	PRGF gradually reduced the TNFα secretion in a dosage dependent manner. PRGF significantly increased the IL-10 secretion with respect to both M0 and M1 macrophages. TNFα, IL-1β, and IL-6 expression decreased by the treatment with PRGF for 24 h. Results depicted an increase in CD206 expression levels after incubation with M1-induced macrophages and PRGF in comparison to only M1-induction or M0 culture media. The pro-inflammatory cytokine markers for TNFα, IL-1β, and IL-6 were reduced in the PRGF-treated nerve compared to the saline control group. The number of pro-inflammatory M1 macrophages was reduced in PRGF-treated groups as indicated by the decrease in CD-68 positive cells.
Cao et al., 2023 (54)	*In vitro*	Bone regeneration	RAW264.7 (BALB/c)	**Group I:** GA **Group II:** PRP-GA **Group III:** PRP-GA@Lap	Towards M2	The expression of iNOS and CCR7 (M1 marker) genes was decreased in cells cultured on PRP-GA and PRP-GA@Lap hydrogels compared to the pure GA group, while the expression of Arg1 and CD206 (M2 marker) genes was increased. These results demonstrated that both sets of hydrogels containing PRP could promote macrophage polarization to M2.
Kargarpour et al. 2021 (61)	*In vitro*	Bone regeneration	RAW 264.7, primary macrophages from murine bone marrow (BALB/c) and osteoclasts from bone marrow cultures	**Group I:** WO (unstimulated cells) **Group II:** PPP **Group III**: BC **Group IV**: Alb-gel **Group V:** RC- in the presence or absence of different TLR agonists - for a more exhaustive treatments' description, check the article directly	Towards M2	BC and PPP lysates inhibited the inflammatory response of macrophages exposed to LPS that goes along with a reduced p65 phosphorylation and NFκB nuclear translocation. Same results were obtained when macrophages were stimulated with another TLR4 (lactoferrin), and poly (1:C) HMW and imiquimod, agonists of TLR3 and TLR7, respectively. Lysates of BC and PPP induced an M1-to-M2 polarization switch. They increased the expression of ARG1 and YM1 (M2 markers). Lysates of the BC, PPP and RC reduced the formation of osteoclasts *in vitro*. The inhibition of osteoclastogenesis was not necessarily caused by platelets, but more likely by parts of the PPP that were also present in BC. The lysates of Alb-gel were considerably less potent to lower the LPS and lactoferrin-induced IL6 expression. The molecules responsible for the anti-inflammatory activity of PPP may be heat sensitive.
Hudgens et al. 2016 (59)	*In vitro*	Tendon regeneration	Rat resident peritoneal macrophages	**Group I:** PPP **Group II:** PRP	No modulate macrophage polarization	PRP resulted in an induction in the expression of the M1 proinflammatory markers iNOS, IL1β and VEGF, with no changes in the expression of CCR7, CD11b, CD68, IL15 or TNFα. There was also a modest induction in several M2 antiinflammatory markers including arginase 1, IL10, CD163 and CD14, with no change in FGF2, CD206, CD168, TGFβ or IGF1 expression. PRP did not appear to significantly modulate macrophage polarization.
Nasirzade et al. 2020 (64)	*In vitro*	Tissue regeneration	Murine bone marrow derived macrophages and RAW 264.7 (BALB/c)	**Group I:** control **Group II:** saliva **Group III**: LPS **Group IV**: saliva + PRF **Group V:** LPS + PRF **Group VI:** PRF CM **Group VII:** saliva + PRF CM **Group VIII:** IL-4 **Group IX**: IL-4 + PRF **Group X**: saliva + PRF + SB431542 **Group XI**: PRF + SB431542	Towards M2	PRF lysates and PRF conditioned medium decreased the inflammatory response of macrophages. They increased ARG1 and YM1 expression, suggesting a shift from M1 toward M2 polarization. PRF increased the expression of lipoxygenases. The transition between M1 and M2 occurred partially due to an activation of TGF-β in PRF. The presence of PRF lysates strongly reduced the NF-κB p65 signalling activation.
Ulivi et al. 2014 (75)	*In vitro*	Tissue regeneration	BM-derived macrophages were isolated from C57Bl/6 mice	**Group I**: CTR **Group II**: CM **Group III:** PL-CM **Group IV**: IL-1-CM **Group V:** PL+IL-1-CM In some experiments, neutralizing antibodies were also used.	Proinflammatory	The effect observed in PL-treated MSCs was essentially proinflammatory, increasing the inflammatory response induced by IL-1, leading to activation of NF-kB and production of a large amount of PGE2, but also, among other factors, to an increase of GM-CSF. Conditioned media of PL-treated MSCs induced in macrophages the expression of GM-CSF and TNF-α keeping them in a proinflammatory phenotype.
Li et al. 2022 (62)	*In vitro* and *in vivo*	Bone regeneration	RAW 264.7 (BALB/c)	**Group I**: Control **Group II**: i-PRF **Group III**: PnP NFs **Group IV:** PnP i-PRF	Moderate and unsustainable M2 macrophage induction effect	Immunofluorescent imaging of CD206 and iNOS indicated that the PnP-iPRF hydrogels could effectively induce M2 macrophage activation in a sustained manner, which could be attributed to the immunomodulatory activity of the PDA component in the PnP-iPRF hydrogels. The i-PRF hydrogels showed moderate and unsustainable M2 macrophage induction effect. Continuous increases in the gene expressions of M2-related markers (CD206, Arg-1, and IL-10) could be observed in the group of PnP-iPRF hydrogels, with concomitant decreased expressions of M1-related markers (iNOS, TNF-α, and IL-6). The cytokines secreted by PnP-iPRF-polarized M2 macrophages could effectively promote osteoblastic differentiation of BMSCs. CD206 and iNOS demonstrated that the PnP-iPRF hydrogels could effectively induce M2 macrophage activation at 4 weeks postsurgery for bone regeneration *in vivo*.
Jiang et al. 2021 (60)	*In vitro* and *in vivo*	Osteochondral regeneration	RAW264.7 (BALB/c)	*In vitro:* **Group I:** control (plastic well) **Group II**: GelMA **Group III**: GelMA + PRP *In vivo:* **Group I**: Normal **Group II**: Sham **Group III**: GelMA **Group IV**: GelMA + PRP	Towards M2	The cells cultured on 20% PRP-GelMA hydrogel exhibited reduced expression of IL-1 β, IL-6, INOS, and CCR7 (M1 marker) genes as compared to the control and pure GelMA groups. The expression of Arg1, IL-1ra, IL-10, and CD206 (M2 marker) genes was higher in the PRP-GelMA group than in the other groups. No significant difference was observed between the pure GelMA and control group. *In vivo*, at 6 weeks, Arg1 and CD163 in the PRP-GelMA group were more positive than in the other groups. After 12 weeks, no significant difference in Arg1 was found among the 3 groups. At 18 weeks, a decrease in the Arg1 content in the PRP-GelMA group reached a lower level than that in the other groups. The staining intensity of CCR7 in the PRP-GelMA group decreased to the lowest level among the 3 groups at 18 weeks.
Qian et al. 2022 (70)	*In vitro* and *in vivo*	Intervertebral disc degeneration	BMDMs from SD rats	*In vitro:* **Group I**: Control **Group II**: PRP **Group III**: PRP-Exos These are the main treatments; interactions with other molecules such as LPS, TRAF-6, IL4 are also included. For a more exhaustive treatments' description, check the article directly. *In vivo:* **Group I**: Control **Group II**: Model **Group III**: Model + PRP **Group IV**: Model + PRP-Exos	Towards M2	PRP and PRP-derived exosomes inhibited the expression of M1-related genes (Il-1b, Il-6, Tnfα, Inos, Il-12 and Pge2) in a dose-dependent manner. PRP-derived exosomes inhibited M1 macrophage polarization by inactivating the NF-κB and MAPK pathways and targeting TRAF6. PRP-derived exosomes increased the expressions of M2-type macrophage-related genes (Arg-1, CD206, CD163 and IL-10) compared with PRP, thus promoting the polarization of M2 macrophages through the STAT6 pathway. The inhibitory effects of the exosomes on IL-1β and Caspase1 in cell supernatant were stronger than in PRP. PRP-derived exosomes promoted the autophagic degradation of NLRP3 by increasing NLRP3 ubiquitination and reducing IL-1β and Caspase-1 production. The expression of inflammasome-related proteins (IL-1β, Caspase1 and NLRP3) was reduced in the PRP-derived exosomes group *in vivo*.
Tang et al. 2022 (72)	*In vitro* and *in vivo*	Tissue regeneration	RAW 264.7 (BALB/c)	**Group I:** ctr (PBS) **Group II**: t-PRP **Group III:** DAT **Group IV:** t-DPI	Towards M2	The t-DPI hydrogel group demonstrated the highest staining intensity of CD206 compared to the DAT hydrogel and t-PRP groups. The results of qPCR on M2 macrophage markers (Arg1, Fizz1, Ym1) further demonstrated these trends. *In vivo*, the percentage of M2 macrophages (F4/80 and CD206) in the t-DPI hydrogel was significantly higher than those of the t-PRP and DAT hydrogel groups. The t-DPI hydrogel promoted the M2 macrophage polarization *in vivo* owing to the t-PRP and DAT in the hydrogel.
Zhang et al. 2020 (79)	*In vitro* and *in vivo*	Tissue regeneration	RAW264.7 (BALB/c)	*In vitro:* **Group I:** WB **Group II**: i-PRF *In vivo:* **Group I:** control **Group II**: i-PRF	Towards M2	TNFα and IL-6 expressions decreased due to suppression, whereas anti-inflammatory M2-polarized macrophage phenotype-associated cytokines (ARG1 and CD206) expressions increased because of activation with i-PRF compared with WB. i-PRF inhibited p65 phosphorylation and expression of TLR4. iNOS2 and ARG1 expressions also changed correspondingly. i-PRF suppressed macrophage M1 polarization by altering the expression of costimulatory molecules and inflammatory cytokines. *In vivo*, there was less inflammatory cells infiltration in i-PRF groups. Adding i-PRF dramatically decreased the amount of local innate immune cells.
Zhao et al. 2022 (80)	*In vitro* and *in vivo*	Tissue regeneration	RAW264.7 (BALB/c)	*In vitro* and *in vivo*: **Group I:** control **Group II:** AG **Group III:** AG-5P	Towards M2	The group of AG-5P showed less staining of iNOS and more staining of Arg-1 than the control and AG groups. *In vivo*, the lowest levels of CD3, CD68, and MPO were detected in the AG-5P groups compared with control and AG group, suggesting little sign of inflammation. There was a decreased number of iNOS^+^ cells and an increased number of Arg-1^+^ cells in the AG-5P group over the control and AG group. PRP incorporation significantly reduced macrophage infiltration into the 3D printed substitutes and promoted the polarization from M1 to M2 type macrophages.
Qian et al. 2022 (69)	*In vivo*	Cardiovascular diseases	N/A (from rats)	**Group I:** MI **Group II:** ALG-HA **Group III:** ALG-HA (PRP) **Group IV:** ALG-HA (Ly-PRF)	Towards M2	The ALG-HA (Ly-PRF) group showed a significantly lower number of iNOS-positive cells and a significantly higher number of CD163-positive cells within the IZ than the MI, ALG-HA, and ALG-HA (PRP) groups, respectively. Similarly, fewer iNOS-positive cells were present in the BZ of the ALG-HA (Ly-PRF) group than MI, ALG-HA, and ALG-HA (PRP) groups. The ALG-HA (Ly-PRF) group also showed a significantly increased number of CD163-positive cells in the BZ versus other groups. Ly-PRF reduced the total number of M1 macrophages and shifted the macrophage polarization towards the M2 phenotype, hence improved the immunoreaction in conditions of MI, which might contribute to the downstream regulation of myocardial fibrosis. Ly-PRF hydrogel might act as a protective unit limiting the loss of wound-healing macrophages.
Park et al. 2021 (68)	*In vivo*	Skin repair	N/A (SD rats)	**Group I:** origin **Group II:** UVR-NS (normal saline) **Group III:** UVR-PRP	Towards M2	Seven days after UVR treatment, M1 macrophage–related molecules were overexpressed, while M2 macrophage–related molecules were under-expressed in the PRP group to promote inflammation. The opposite results were observed 28 days after treatment in the PRP group to inhibit inflammation and promote repair. The result of the ACVR IIA–FST system was consistent with the tendency of macrophages to polarize. PRP played an important regulatory role in helping reduce UVB-induced acute skin tissue inflammation by adjusting macrophage polarization, which alleviated skin inflammation and stimulated collagen regeneration.
Nishio et al. 2020 (65)	*In vivo*	Tendon healing	N/A (C57BL/6 and B6.129P-Cx3cr1^tm1Litt^/J)	**Group I:** control **Group II:** LP-PRP **Group III:** LR-PRP	Both (LR-PRP: M1; LP-PRP: M2)	The tendon healing was significantly earlier in LP-PRP group than those of LR-PRP group on postoperative day 28. The number of M1 in control group was highest, but not statistically significant, on postoperative day 28, while those in the LR-PRP and LP-PRP groups were significantly highest at day 4 and decreased with time. The number of M2 in control group was highest, but not statistically significant, on postoperative day 28, while in the LR-PRP and LP-PRP groups were highest on postoperative day 7, the latter significantly. In control group, M1/M2 ratio was significantly highest on postoperative day 14, while those in LP- and LR-PRP was highest on postoperative day 1, but not statistically significant. The ratio of M1/M2 was below 1.0 only in LP-PRP groups at day 7 and 14. Both LP- and LR-PRP enhanced the recruitment of MPs but LR-PRP mainly enhanced the effects of M1 MPs, whereas LP-PRP more strongly induced the activity of M2 MPs.
Yu et al. 2021 (78)	*In vivo*	Tendon healing	N/A (SD Rat)	**Group I**: PRPr **Group II:** control (saline solution)	Antiinflammatory	The fraction of ED1+ macrophages was higher in the saline as compared with the PRPr group. On day 10 postinjury, the percentage of ED1+ macrophages was reduced in both groups, with no difference between them. PRP dampened down the ED1+ macrophage–related inflammation just enough to create an ideal tendon healing environment that might contribute to tendon cell proliferation and inhibit cell apoptosis and collagen degradation.
Wessely-Szponder et al. 2019 (76)	*In vitro*	Tissue regeneration	Ovine monocyte-derived macrophage	**Group I:** BCS **Group II:** LPS **Group III**: DEX **Group IV**: PRP **Group V:** LPS+PRP **Group VI:** DEX+PRP **Group VII:** AMP **Group VIII:** LPS + AMP **Group IX**: DEX + AMP	Towards M1	After contact with PRP, Mfs changed towards the pro-inflammatory response both in cultures after previous stimulation with LPS and in those without this stimulation. The stimulation of Mfs with PRP resulted in more superoxide generation, with the highest response evident after priming with LPS. The experiment revealed more powerful generation of NO in cultures stimulated with PRP alone or in combination with LPS (LPS + PRP) or DEX (DEX + PRP). Arginase activity significantly decreased after addition of LPS, PRP, LPS+PRP or AMP to Mf cultures, whereas previous stimulation with DEX caused a slight increase in arginase activity. Previous addition of DEX to Mfs cultures weakened the pro-inflammatory response of Mfs to PRP. Both, AMP and PRP shift Mfs towards a pro-inflammatory rather than a repair phenotype.

ACVR2A, activin receptor type-2A; AG, alginate-gelatin; AG-5P, AG with 5% PRP; AKT, phosphatidylinositol 3-kinase (PI3K)/protein kinase B; Alb-gel, albumin gel; ALG-HA, alginate and hyaluronic acid; AMP, antimicrobial peptides; APS, autologous protein solution kit; Arg1, arginase 1; BC, buffy coat; BCS, bovine calf serum; BMDMs, bone marrow-derived macrophages; BMSCs, bone marrow mesenchymal stem cells; BZ, border zone; CCM, CGF-conditioned medium; CCR7, C-C chemokine receptor type 7; CGF, concentrated growth factor; CM, conditioned medium; CTR, serum-free standard médium; CXCL10, C-X-C Motif Chemokine Ligand 10; DAT, decellularized adipose tissue; DC-LD, dendritic cells treated with leukodepleted PRP; DC-ST, dendritic cells treated with standard PRP; DEX, dexamethasone; Exos, exosomes; FBS, fetal bovine serum; FCS, fetal calf serum; FGF2, fibroblast growth factor 2; Fizz1, resistin-like molecule alpha1; FST, follistatin; GA, methacrylated gelatin (GelMA) and methacrylated alginate (AlgMA); GelMA, gelatin methacryloyl; GM-CSF, granulocyte-monocyte colony stimulating factor; hPL, human platelet lysate; hS, human serum; iDC, immature dendritic cells; IFNγ, interferon gamma; IGF1, insulin-like growth factor 1; IL-10, interleukin-10; IL-12, interleukin-12; IL-15, interleukin-15; IL-15, interleukin-15; IL-1ra, interleukin-1 receptor antagonist; IL-1β, interleukin-1β; IL-4, interleukin-4; IL-6, interleukin-6; IL-8, interleukin-8; iNOS, inducible nitric oxide synthase; i-PRF, injectable PRF; IZ, infarcted zone; JPCs, jaw periosteal cells; LP-PRP, leukocyte-poor PRP; LP-PRP, leukocyte-poor PRP; LPS, lipopolysaccharide; LR-PRP, leukocyte-rich PRP; Ly-PRF, lyophilized PRF; MAPK, mitogen-activated protein kinase; M-CSF, macrophage colony-stimulating factor; MDM, monocyte derived macrophage; mEVs, medium extracellular vesicles; Mfs, macrophages; MI, myocardial infarction; MPO, myeloperoxidase; MPs, macrophages; MSCs, mesenchymal stem cells; N/A, not applicable; N.S., not specified; NFκB, the nuclear factor kappa B; NLRP3, NOD-, LRR- and pyrin domain-containing protein 3; NO, nitric oxide; OA, osteoarthritis; PBMC, peripheral blood mononuclear cells; PBS, phosphate-buffered saline; PDA, polydopamine; PGE2, prostaglandin E2; PL, platelet lysate; PnP NFs, PDA-functionalized (PCL/nHA) nanofibers; PPAR, peroxisome proliferator-activated receptors; PPP, platelet-poor plasma; P-PRP, pure platelet-rich plasma; PRF CM, PRF conditioned medium; PRF, platelet-rich fibrin; PRGF, platelet-rich growth factors; PRP, platelet-rich plasma; PRP-GA@Lap, PRP-GA and laponite hydrogel; PRP-LD, leucodepleted; PRPr, PRP releasate; PRP-ST, standard PRP; qPCR, quantitative polymerase chain reaction; RANKL, receptor activator of nuclear factor-kappaB ligand; RC, red clot; SB431542, the inhibitor of TGF-β receptor type I kinase; SD rats, Sprague Dawley rats; sEVs, small extracellular vesicles; S-PRP, supernatant of calcium-activated P-PRP; STAT6, signal transducer and activator of transcription 6; suppl, supplement; t-DPI, the thermosensitive decellularized adipose tissue/platelet-rich plasma interpenetrating polymer network; TGF-β, transforming growth factor beta; THP-1, human monocytic cell line; TLR, toll-like receptors; TNF-α, tumor necrosis factor-alpha; t-PRP, temperature-controlled PRP; TRAF6, tumor necrosis factor receptor associated factor 6; UVR, ultraviolet radiation; VEGF, vascular endothelial growth factor; w/, with; w/o, without; WB, whole blood; WO, without (unstimulated cells); YM1, a rodent-specific chitinase-like protein.

### Reporting quality and risk of bias

**Figure 2:**
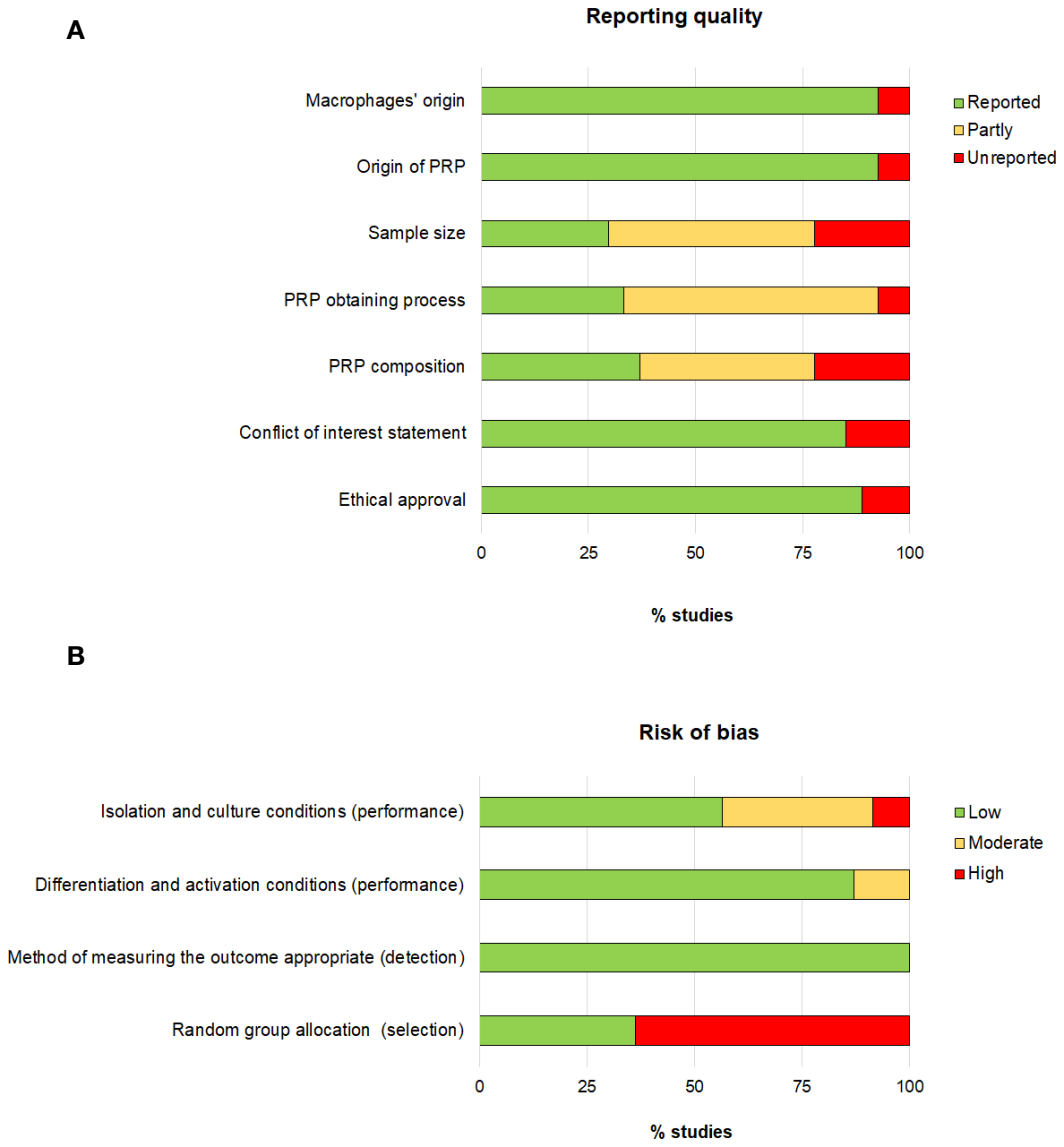
Assessment of the reporting quality **(A)** and risk of bias **(B)**.

The reporting quality showed large differences (**Figure 2**). The lack of information regarding sample size, PRP obtaining process and PRP composition had the greatest impact on the reporting quality as 70%, 66% and 63% of the studies, respectively, did provide partial or no information on these issues. Concerning the risk of bias, the risk associated with the selection bias was the highest detected risk, as only 36% of the articles did specify that animals were randomly assigned.

### Platelet derivatives

**Figure 3:**
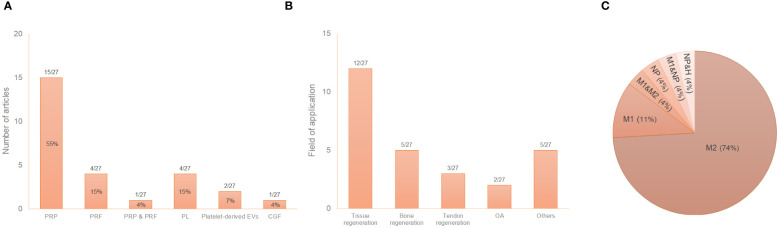
Distribution of the selected publications according to the type of platelet derivative **(A)**, field of application **(B)** and type of induced macrophage polarization (**C** ^*^). ^*^The graph reflects situations where two different states were reported in the same study. GraÇa et al. (57) reported a hybrid M1/M2 phenotype and a non-polarized state depending on the product studied. Tylek et al. (73) reported both non-polarized state and M1-like type according to the study conditions. Nishio et al. (65) showed polarization towards both phenotypes (M1 and M2) depending on the platelet derivative. NP, non-polarized state; H, hybrid phenotype.

The great variability of PRP composition and preparation protocols leads to a myriad of biologically distinct products. This issue along with the lack of information in the literature, makes challenging the comparison of results (81, 82). In fact, details such as the sample size, the type of anticoagulant and activator, centrifugation conditions or the composition were often missing in this review. Fifty-nine percent of the articles used PRP in their assays compared to 19% and 15% that used PRF or PL, respectively (**Figure 3A**). **Table 2** describes the conditions for obtaining the different platelet derivatives. In only 8 out 27 articles (55, 56, 64, 65, 72, 77–79) were these conditions fully defined. Of those, Cenni et al. (55), Escobar et al. (56), Nishio et al. (65) and Yu et al. (78) completely detailed the cellular composition which was also fully described in other articles (60, 66, 67, 74, 75, 80) where the obtaining protocol was not totally detailed. The origin was human or murine except in two of the articles that PRP from rabbit and mouse (60) or ovine (76) was used. Most used double centrifugation (n = 14) versus single (n = 6). Qian et al. (69) even used both centrifugation methods depending on the type of platelet derivative to be obtained. However, in this regard, conditions such as centrifugal force, speed and time were extremely varied, with as many different protocols as studies. Concerning the cellular composition, 33% of the articles did not provide information about the presence of leukocytes in their studies. Among those that did detail this issue (presence of leukocytes), the majority did not include leukocytes (n = 10), compared to the others (n=6) in which the leukocytes were included. Moreover, the study proposed by Uchiyama et al. (74) was the only one that had two types of platelet derivatives tested, with and without leukocytes, while Kargarpour et al. (61) evaluated several fractions from liquid PRF containing different composition.

**Table 2:** Description of the obtaining process of the platelet derivatives from the reviewed articles. Sample size and leukocyte inclusion are also detailed.

Ref.	Type	Origin	Sample size (donors)	Anticoagulant	Centrifugation (force / time)	Leukocytes	Activator
Cenni et al., 2010 (55)	PPP and PRP	Human	N.S.	Sodium citrate	Double 1ª: 180g / 5 min 2ª: 600g / 15 min	No	Bovine thrombin and calcium gluconate
He et al. 2021 (58)	PL	Human	N.S.	N.S.	N/A	No	N.S.
O’Donnell et al. 2019 (66)	PRP	Human	19	N.S.	Double 1ª: 1000 rpm / 10 min 2ª: 800 rpm / 9 min	No	N.S.
Uchiyama et al. 2021 (74)	LP-PRP (Cellaid Serum Collection Set P type kit) and APS, LR-PRP (Autologous Protein Solution kit) - Codes: 210-00-00 and 214-15-10, respectively*	Human	12	ACD-A	LP-PRP: Double 1ª: 200 g / 15 min 2ª: 1200 g / 15 min APS: Double 1ª: 745 g / 15 min 2ª: 219 g / 2 min	LP-PRP: no; APS, LR-PRP: yes	LP-PRP: none; APS: yes but N.S.
Escobar et al., 2018 (56)	P-PRP and S-PRP	Human	15	Sodium citrate	Single 100 g / 5 min	No	P-PRP: none S-PRP:CaCl_2_
GraÇa et al. 2022 (57)	Platelet-derived EVs	Human	Each unit of platelets from 5 donors / 15–16 platelet concentrates	N.S.	N/A	No	Freeze/ thaw cycles
Luo et al. 2021 (63)	CGF	Human	10	None	A special program as follows: 30 s acceleration, 2 min at 2700 rpm, 4 min at 2400 rpm, 4 min at 2700 rpm, 3 min at 3000 rpm and 36 s deceleration	Yes	N.S.
Papait et al. 2018 (67)	PRP (PRP-ST and PRP-LD)	Human	N.S.	N.S.	Double 1ª: 388 rcf / 10 min 2ª: 2169 rcf / 20 min	No	N.S.
Scopelliti et al. 2021 (71)	PL (Euroclone)	N.S.	N.S.	N.S.	N.S.	N.S.	N.S.
Tylek et al. 2019 (73)	PL (PL Bioscience)	Human	N.S.	N.S.	N.S.	No	N.S.
Yadav et al. 2022 (77)	PRGF	SD rats	N.S.	Sodium citrate	Single 1900 rpm / 8 min	No	CaCl_2_
Cao et al., 2023 (54)	PRP	Rat	N.S.	Heparin	Double N.S.	N.S.	CaCl_2_
Kargarpour et al. 2021 (64)	Liquid PRF	Human	6	None	Single 2000 g / 8 min	**	Dual freeze-thawing, followed by sonication
Hudgens et al. 2016 (59)	PPP and PRP	Rat	N.S.	Sodium citrate	Double 1ª: 500 g / 5 min 2ª: 700 g / 17 min	N.S.	N.S.
Nasirzade et al. 2020 (64)	PRF (PRF lysates and PRF CM)	Human	6	None	Single (1570 rpm / 12 min)	N.S.	Freeze-thawing and sonication
Ulivi et al. 2014 (75)	PL	Human	At least 10	N.S.	N.S.	No	Freeze-thaw cycles
Li et al. 2022 (62)	i-PRF	In vitro: human In vivo: rats	In vitro: 8 In vivo: N.S.	None	Single 700 rpm / 3 min	N.S.	N.S.
Jiang et al. 2021 (60)	Lr-PRP	Rabbit and mouse	N.S.	N.S.	Double 1ª: 200 g / 10 min 2ª: 600 g / 8 min	Yes	N.S.
Qian et al. 2022 (70)	PRP-derived exosomes	SD rats	N.S.	Sodium citrate	Double 1ª: 377 g / 10 min 2ª: 377 g / 10 min	N.S.	N.S.
Tang et al. 2022 (72)	t-PRP	Human	N.S.	None	Double 1ª: 200 g / 10 min 2ª: 1550 g / 10 min	N.S.	Temperature
Zhang et al. 2020 (79)	i-PRF	Wistar rats	8	None	Single 700 rpm / 3 min	Yes	None
Zhao et al. 2022 (80)	PRP	Human and SD rats	N.S.	Sodium citrate	Double 1ª: 900 g / 5 min 2ª: 1500 g / 15 min	Yes	N.S.
Qian et al. 2022 (69)	PRP and Ly-PRF	N.S.	N.S.	PRF: None PRP: N.S.	PRP: Double 1ª: 45 g / 7 min 2ª: 400 g / 10 min Ly-PRF: Single 3000 rpm / 10 min	N.S.	PRF: None PRP: N.S.
Park et al. 2022 (34)	PRP	SD rats	8	Sodium citrate	Double 1ª: 300 g / 5 min 2ª: 700 g / 17 min	Yes	N.S.
Nishio et al. 2020 (65)	PRP (Tornado-N technique)	Mouse	40	EDTA-2Na	Double 1ª: 220g / 10 min 2ª: 2400g / 10 min	Yes	Calcium chloride
Yu et al. 2021 (78)	PRPr	SD rats	10	Acid citrate dextrose solution	Double 1ª: 800 g / 20 min 2ª: 3000 g / 20 min	No***	Thrombin solution
Wessely-Szponder et al. 2019 (76)	PRP	Ovine	N.S.	CPDA	Double 1ª: 160 g / 20 min 2ª: 400 g / 15 min	N.S.	N.S.

* According to Kon et al. 2020 (83).

** Depending on the fraction. PPP and Alb-gel: no leukocytes; BC: leukocytes; RC: N.S.

*** If leukocyte inclusion is considered when values are above the baseline of whole blood. They reported a concentration of 0.8-fold over baseline.

ACD-A, anticoagulant citrate-dextrose solution A; APS, autologous protein solution kit; CGF, concentrated growth factor; CPDA, citrate phosphate, dextrose, and adenosine; EDTA, ethylenediaminetetraacetic acid; EVs, extracellular vesicles; G, g-force; i-PRF, injectable PRF; LP-PRP, leukocyte-poor PRP; LR-PRP, leukocyte-rich PRP; Lr-PRP, leukocyte-rich PRP; Ly-PRF, lyophilized PRF; N.S., not specified; N/A, not applicable; PL, platelet lysate; PPP, platelet-poor plasma; P-PRP, pure platelet-rich plasma; PRF CM, PRF conditioned medium; PRF, platelet-rich fibrin; PRGF, platelet-rich growth factors; PRP, platelet-rich plasma; PRP-LD, leucodepleted PRP; PRPr, PRP releasate; PRP-ST, standard PRP; RCF, Relative centrifugal force; RPM, revolutions per minute; SD rats, Sprague Dawley rats; S-PRP, supernatant of calcium-activated P-PRP; t-PRP, temperature-controlled PRP.

### Macrophages

As reflected in the data shown in **Table 1**, most of the studies used murine macrophages (n = 15), compared to 41% that used human macrophages (n = 11). Only one article used macrophages of ovine origin (76). Regarding the type of macrophages in *in vitro* studies, tissue resident macrophages were used in only two articles (59, 61) compared to the rest of the studies that used monocyte derived macrophages. Macrophage isolation, culture, differentiation and polarization protocols greatly differed among studies (**Table 3**). The immunoregulatory effect of platelet derivatives was assessed in several fields of application such as tissue, bone and tendon regeneration, osteoarthritis, cardiovascular diseases, intervertebral disc degeneration, nerve and osteochondral regeneration and skin repair (**Figure 3B**). Although the reported results being diverse, the treatment with platelet derivatives stimulated macrophages to exhibit a reparative phenotype in most of the studies (n = 21) (**Figure 3C**).

**Table 3:** Summary information of the macrophage differentiation and polarization protocols *in vitro*.

Reference	Origin	Isolation	Culture medium*	Initial density	Differentiation and activation factors	Differentiation and activation time	Resting period**
Cenni et al., 2010 (55)	Osteoclasts derived from human PBMC	Ficoll-Hystopaque (Density gradient)	DMEM high glucose	3 x 10^6^ cells/cm^2^	Osteoclasts: 30 ng/mL RANKL + 25 ng/mL M-CSF	7, 10 and 9 days according to the assays	No
He et al. 2021 (58)	THP-1	N/A	RPMI 1640 + 0.05 nM 2-mercaptoethanol	6 x 10^5^ cells/well	M0: 5 nM PMA M1: 15 ng/mL LPS + 20 ng/mL IFNγ M2: 20 ng/mL IL-13 + 20 ng/mL IL-4	M0: 48h M1 and M2: 24h and 72h	No
O’Donnell et al. 2019 (66)	Human PBMC	Ficoll-Paque (Density gradient)	RPMI 1640	2.5 × 10^5^ cells/well	M0: 30 ng/mL M-CSF M1: 50 ng/mL LPS	M0: 7days M1: 24h	No
Uchiyama et al. 2021 (74)	Human PBMC	Density gradient (Histopaque)	RPMI 1640 + GlutaMAX^TM^	1 x 10^5^ cells/cm^2^	M0: 20 ng/mL M-CSF M1: 100 ng/mL LPS + 50 ng/mL IFNγ M2: 20 ng/mL IL-4	M0: 6 days M1 and M2: 2 days	No
Escobar et al., 2018 (56)	Human PBMC	RosetteSep™ Human Monocyte Enrichment Cocktail (Immunodensity negative selection cocktail)	RPMI 1640	3 x 10^6^ cells/well	M0: 50 ng/mL M-CSF M1: 100 ng/mL LPS + 20 ng/mL IFNγ	M0: 7 days M1: 24h	No
GraÇa et al. 2022 (57)	THP-1	N/A	RPMI 1640 + GlutaMAX^TM^	N.S.	M0: 100 nM PMA	M0: 3 days	Yes (Washed with fresh medium and cultured for 48 h)
Luo et al. 2021 (63)	THP-1	N/A	RPMI 1640	1 x 10^6^ cells/mL	M0: 100 ng/mL PMA	M0: 24h	No
Papait et al. 2018 (67)	Human PBMC	Ficoll-Hystopaque (Density gradient) and RosetteSep™ Human Monocyte Enrichment Cocktail	RPMI 1640 + 1% L-glutamine	2.5 x 10^5^ cells/well	M0: none DC: 20 ng/mL IL-4 + 20 ng/mL GM-CSF	M0: N.S. DC: 6 days	No
Scopelliti et al. 2021 (71)	Human PBMC	Lympholyte-H (Density gradient) and immunomagnetic beads	RPMI 1640 + 200 mM glutamine	N.S.	M0: 10 ng/mL M-CSF M1: 100 ng/mL LPS + 10 ng/mL IFNγ	M0: 6 days M1: 48h	No
Tylek et al. 2019 (73)	Human PBMC	Pancoll (Density gradient) and Pan Monocyte Isolation Kit	RPMI 1640 + GlutaMAX^TM^	N.S.	M0: none M1: 1 µg/mL LPS M2: 10^-7^ M dexamethasone	M0: up to 7 days M1 and M2: 7 days	No
Yadav et al. 2022 (77)	THP-1	N/A	RPMI	1 x 10^5^ cells/mL	M0: 150 nM PMA M1: LPS + IFNγ	M0: 24h M1: 24h	Yes (Washed with PBS to eliminate the PMA and grew in RPMI media for 24h)
Cao et al., 2023 (54)	RAW264.7 (BALB/c)	N/A	DMEM	4 x 10^3^	M1: 100 ng/mL LPS	M1: 8h	N/A
Kargarpour et al. 2021 (64)	RAW 264.7, primary macrophages from murine bone marrow (BALB/c) and osteoclast from bone marrow cultures	RAW 264.7: N/A Bone marrow macrophages: N.S. Osteoclasts: N.S.	RAW 264.7 and Bone marrow macrophages: DMEM Osteoclasts: αMEM	RAW 264.7: 2 × 10^5^ cells/cm^2^ Bone marrow macrophages: 4 × 10^6^ cells/cm^2^ Osteoclasts: 4 × 10^6^ cells/cm^2^	Bone marrow macrophages: M0: 20 ng/mL M-CSF RAW 264.7 and Bone marrow macrophages: M1: 100 ng/mL LPS or 50 ng/mL lactoferrin or 10 µg/mL poly (1:C) HMW or 5 µg/mL imiquimod M2: 120 ng/mL IL-4 Osteoclasts: 30 ng/mL RANK-L + 20 ng/mL M-CSF + 10 ng/mL TGF-β1	Bone marrow macrophages: M0: 5 days RAW 264.7 and Bone marrow macrophages: M1 and M2: 24h Osteoclasts: 6 days	No
Hudgens et al. 2016 (59)	Rat resident peritoneal macrophages	N/A	N.S.	N.S.	GM-CSF	N.S.	N/A
Nasirzade et al. 2020 (64)	Murine bone marrow derived macrophages and RAW 264.7 (BALB/c)	Bone marrow macrophages: N.S. RAW 264.7: N/A	αMEM	Bone marrow macrophages: 4 x 10^6^ cells/cm^2^ RAW 264.7: 1 × 10^5^ cells/cm^2^	Bone marrow macrophages: M0: 20 ng/mL M-CSF Both types: M1: 5% saliva + 100 ng/mL LPS M2: 10 ng/mL IL-4	Bone marrow macrophages: M0: 5 days Both types: M1 and M2: overnight	No
Ulivi et al. 2014 (75)	BM-derived macrophages were isolated from C57Bl/6 mice	The supernatant containing the cells that did not adhere was collected and replated in a 150-mm ‘‘non-treated’’ culture dish	αMEM + 2mM L-glutamine + 25 µg/mL GM-CSF	N.S.	M0: 25 µg/mL GM-CSF	M0: 5 days	No
Li et al. 2022 (62)	RAW 264.7 (BALB/c)	N/A	DMEM	N.S.	None	M2: 48h	N/A
Jiang et al. 2021 (60)	RAW264.7 (BALB/c)	N/A	DMEM 4.5 g/l D-glucose	3 x 10^3^ cells/well	M1: 100 ng/mL LPS	M1: 8h	N/A
Qian et al. 2022 (70)	BMDMs from SD rats	Centrifuged at 4 °C and 600 g for 5 min. The supernatant was discarded	RPMI 1640	1 x 10^6^ cells/well	M0: 10 ng/mL M-CSF M1: 200 ng/mL LPS M2: 20 ng/mL IL-4	M0: 5 days M1 and M2: 24h	Yes (Culture medium was changed to DMEM containing 1% FBS media at 16h before treatment)
Tang et al. 2022 (72)	RAW 264.7 (BALB/c)	N/A	N.S.	5 x 10^4^ cells/well	None	M2: 48h	N/A
Zhang et al. 2020 (79)	RAW264.7 (BALB/c)	N/A	DMEM	1 × 10^5^ cells	M1: 100 ng/mL LPS	M1: 24h	N/A
Zhao et al. 2022 (80)	RAW264.7 (BALB/c)	N/A	N.S.	N.S.	M1: 100 ng/mL LPS	N.S.	N/A
Wessely-Szponder et al. 2019 (76)	Ovine monocyte-derived macrophage	Lymphoprep (gradient centrifugation)	DMEM	1.0 × 10^6^ cells/mL	M0: none M1: 1 µg/mL LPS M2: 100 nM dexamethasone	M0: 72h M1 and M2: 24 and 48h	No

* The main culture medium and specific supplements are detailed, but the antibiotics and FBS are not listed.

** The resting period refers to whether macrophages rested from the inducer to differentiate prior to activation/polarization.

αMEM, Minimum Essential Medium Eagle-Alpha Modification; BMDMs, bone marrow-derived macrophages; CSF-1, colony stimulating factor 1; DC, dendritic cells; DMEM, Dulbecco's Modified Eagle Medium; FBS, fetal bovine serum; GM-CSF, granulocyte-monocyte colony stimulating factor; IFNγ, interferon gamma; IL-13, interleukin-13; IL-4, interleukin-4; LPS, lipopolysaccharide; M-CSF, macrophage colony-stimulating factor; N.S., not specified; N/A, not applicable; PBMC, peripheral blood mononuclear cells; PBS, phosphate-buffered saline; PMA, phorbol-12-myristate-13-acetate; RANKL, receptor activator of nuclear factor-kappaB ligand; RPMI 1640, Roswell Park Memorial Institute (RPMI) 1640 medium; SD rats, Sprague Dawley rats.

### Human macrophages

Research in human macrophages were performed in 11 articles. In 8 of which a polarization towards the reparative phenotype (M2) was reported after treatment with the platelet derivatives (55, 56, 58, 63, 67, 71, 74, 77). Cenni et al. (55) specified that their PRP impaired osteoclast formation, which is typical of the M2 phenotype. On the contrary, a pro-inflammatory effect was described in only 2 articles (66, 73); however, certain peculiarities must be taken into account, as O´Donnell et al. (66) evaluated a PRP obtained from OA patients and the results reported by Tylek et al. (73) refer to the co-culture of macrophages with mesenchymal stem cells. Both, a hybrid M1/M2 phenotype and a non-polarized state was described by GraÇa et al. (57) according to the type of extracellular vesicle. All the research conducted in this section evaluated the effect of PRP *in vitro*. Only Yadav et al. (77) did it also *in vivo*.

### In vitro

Cenni et al. (55) evaluated the effect of bovine thrombin and calcium gluconate-activated PRP on human osteoclasts. Osteoclastogenesis is a multi-step process that requires the delicate coordination of osteoclast progenitors. In this sense, M-CSF (macrophage colony stimulating factor) promotes proliferation of the osteoclast precursor cells and RANKL (receptor activator of NFκB ligand) controls their differentiation (84, 85). Therefore, the authors analyzed the ability of PRP supernatant to influence on osteoclast precursors, both with and without RANKL and M-CSF. They concluded that PRP showed different effects depending on its concentration. Whereas 10% induced a similar number of tartrate-resistant acid phosphatase (TRACP)-positive multinucleated cells to the positive control, higher concentrations of PRP did not induce the generation of multinucleated cells positive for this enzyme considered as a marker of bone resorption. Nevertheless, even when the multinucleated cells were treated with 10%PRP, the bone resorptive activity was also significantly lower. According to the authors, these results could be explained by the fact that PRP at high concentration could recruit osteoclast precursors but inhibit their differentiation. They also suggested that the transforming growth factor-β (TGF-β), present in platelets, could be one of the factors responsible for this inhibition. Also regarding bone regeneration, He et al. (58) studied the effect of different medium suplementations (10% FBS, 10% human platelet lysate (hPL) and 5% hPL) on THP-1-derived macrophage polarization. They concluded that human platelet lysate was a better choice for M1 and M2 polarization as cells treated with 5% hPL exhibited a more consistent morphology with the expected phenotype. In fact, culturing with 10%FBS or with 5% hPL induced opposite patterns for M1 and M2 macrophages. The authors also analyzed the effect of jaw periosteal cells (JPCs) on macrophage polarization in direct coculture. They concluded that low concentration of hPL enhanced the ability of JPCs to inhibit M1 polarization compared to other supplementations. The authors also detected the recently discovered CD169^+^ macrophages. They concluded that the JPCs’ ability to regulate this macrophage population could be enhanced by a low concentration of hPL.

O´Donnell et al. (66) and Uchiyama et al. (74) conducted their research on osteoarthritis (OA), although with very different approaches. O´Donnell et al. (66) compared leukocyte-poor PRP from healthy young male donors with the PRP from older male patients with severe knee OA. They also grouped the samples according to the concentration of inflammatory mediators and growth factors into high and low. They reported that according to age and disease state the composition of PRP was different, as PRP from older OA patients showed increased levels of inflammatory cytokines and less growth factors and platelets. These differences were reflected in the response of macrophages to treatment with PRP from these two groups. OA-PRP upregulated mRNA for inflammatory proteins in human monocyte-derived macrophages, thus promoting the inflammatory macrophage phenotype. The authors therefore suggested that these two factors (age and disease condition) may influence the bioactivity of PRP and hence their clinical effect. On the other hand, Uchiyama et al. (74), compared two types of PRP purification kits that resulted in two PRP of different composition (leukocyte-poor PRP (LP-PRP) and leukocyte-rich PRP (APS LR-PRP)) on macrophage phenotypes. They found higher concentrations of both M1 and M2 macrophages related factors in APS. The addition of PRP supernatants decreased the expression of M1 macrophage markers, such as IL-1β, TNF-α, CD80 and CD86, when compared to the monocyte derived macrophages alone, while there were no detected differences in the purification kits. Regarding M2 markers, they showed higher expression of MRC1 when macrophages were cultured with both PRP supernatants with regard to negative control, while the gene expression of IL-10 and TGF-β was increased in cells treated with APS or LP-PRP, respectively. In contrast, cell surface markers of the M2 phenotype (CD163 and CD206) were not altered after PRP culture. They also reported that both types of PRP promoted the repolarization of monocyte-derived M1 macrophages to the M2 phenotype; however, according to the authors, they gave rise to different M2 subpopulations. While LP-PRP promoted polarization toward the M2c subset, mainly related to tissue repair, APS promoted towards M2a, related to anti-inflammatory activity. Nevertheless, this information should be interpreted with caution. M2 macrophages further divide into subsets based on their distinct gene expression profiles. However, many of these subpopulations share markers. Thus, M2a, M2c and M2d subsets are characterized by the secretion of IL-10 and TGF-β, making accurate classification difficult (28). The authors stated that an increase in the ratio M1/M2 macrophages leads to a progression in OA disease. Therefore, they concluded that PRP treatment could improve symptoms in these patients by reducing the imbalance of M1/M2 macrophages.

When it comes to tissue regeneration or wound healing, several articles were also included. Both the study groups and the experimental design were also different in all studies. Escobar et al. (56) evaluated the biological effects of two platelet preparations on the phenotype of human monocyte-derived macrophages. They compared a leukocyte-depleted pure platelet-rich plasma without activation (P-PRP) and the supernatant released from that P-PRP after CaCl_2_ activation (S-PRP). Their findings showed that P-PRP and S-PRP generated different profiles of tissue-repair macrophages. Both preparations stimulated the expression of the M2 marker, CD206. In addition, only P-PRP enhanced the production of the potent anti-inflammatory cytokine IL-10. On the other hand, S-PRP also induced higher levels of expression of another M2 marker (CD163) compared with P-PRP; however, the expression of the pro-inflammatory marker CD86 was also enhanced after S-PRP treatment. Those results suggested different clinical regenerative potentials for both platelet preparations. Recent developments in platelet biology have led to new insights. In this regard, the platelet derived extracellular vesicles (EVs) and their role in intercellular communication are of interest (86). In this sense, the work of GraÇa et al. (57) evaluated the effect of two different EV populations (small EVs and medium EVs) derived from platelet lysates. Macrophage responses varied depending on the EV population they were treated with. sEVs induced polarization towards a hybrid M1/M2 phenotype, while mEVs induced a more non-polarized state. The authors stated that hybrid macrophages, which also exist *in vivo*, promote a more native-like extracellular matrix compared to a predominantly M2 macrophage phenotype. They also suggested that this difference in macrophage response to treatment could be useful as an effective strategy in modulating the process of tissue repair by sequentially using sEVs and mEVs in early and later healing stages, respectively. The immunoregulatory role of another platelet concentrate product in macrophage functional activities was also included (63). In this case, the authors tested different concentrations of conditioned medium (CCM) from concentrated growth factor (CGF). They reported reduced secretion of inflammatory cytokines (such as IL-1β) and enhanced chemokine production (such as RANTES) by CGF-stimulated macrophages, thus promoting M2 polarization. The Akt pathway was also determined to be involved in the immunoregulatory role of this concentrated growth factor. Papait el al (67). analyzed the effect of allogenic PRP mismatched for AB0 and Rh antigens on macrophages differentiation and function. Two types of preparations were used, standard (ST) and leucodepleted (LD). Macrophage cultures with GM-CSF, IL-4 and 10% FCS were referred as immature dendritic cells (iDC), while the addition of 5% ST-PRP or LD-PRP to that treatment were referred to as DC-ST or DC-LD, respectively. Combination of GM-CSF and IL-4 is generally used for the differentiation of human monocytes into dendritic cells (DC) (87, 88). The mononuclear phagocyte system (MPS) has been defined as a family of cells comprising monocytes, dendritic cells and macrophages. Their functional and phenotypical characteristics are often overlapping, making the distinction and classification of these cell types truly challenging. In fact, many of the proposed unique markers and functions are shared between cell types. This has added much confusion about their identity and function, opening the debate regarding which subsets represents distinct cell types and which are versions of the same cell type, thus leading to a possible misinterpretation of the results (89, 90). In fact, authors reported difficulty in clearly defining the type of DC induced by PRP. Their findings showed that PRP could inhibit macrophage differentiation to CD1a^+^ iDC; on the contrary, PRP induced type 2 macrophages, as they expressed high levels of CD163 and CD206. Moreover, release of IL-10 and PGE2 was also induced by PRP, even in the presence of LPS. Although both ST-PRP and LD-PRP induced DC with similar features, the authors stated that the inclusion of leukocytes did influence the secretion of those immunoregulatory factors, as more IL-10 and PGE-2 were reported with DC-LD, however, the white cell content was practically nil in both preparations. Taken together, data suggested that PRP promoted a regulatory anti-inflammatory phenotype thus supporting wound healing. The effects of platelet lysate (PL) on macrophages phenotype and functions were also examined in two other studies with different scopes (71, 73). Scopelliti et al. (71) used a commercial PL to conduct their assays in order to limit individual variation; however, as they stated, this also involved a drawback, as information about its composition was also missing. The authors exposed M1-polarized macrophages to 10% PL. They demonstrated that PL treatment reduced the expression of M1 markers (such as CD80 and CD86) and enhanced the expression of M2 markers (such as CD206, CD200R, PPAR and arginase). In addition, TNF-α and NFκB expression was significantly reduced. On the other hand, the release of TGF-β and VEGF, both largely involved in the wound healing process, was significantly increased along with the expression of STAT3, STAT6, SMAD2 and SMAD4. Therefore, the authors concluded that PL repolarized M1 macrophages towards a M2-like phenotype, thus favoring the regenerative process. Tylek et al. (73) evaluated another commercial platelet lysate as an alternative for *in vitro* culture of macrophages as well as for co-culture with hMSCs. The authors concluded that hPL, especially without adding heparin, was the best performing supplement both for the *in vitro* culture of human monocyte-derived macrophages and for the co-culture system, compared to human serum and fetal calf serum.

### *In vitro* and *in vivo*

Finally, Yadav et al. (77) focused their research on nerve regeneration and conducted their assays both *in vitro* and *in vivo*. The authors studied, *in vitro*, the effect of different percentages of PRGF on M0 and M1 macrophage polarization. PRGF treatment inhibited the M1 phenotype. Consistent with Scopelliti et al. (71) they also showed a decrease in TNF-α, in addition to IL-1β and IL-6. However, in contrast to Scopelliti et al., who used a platelet lysate, the PRGF used in this experimental setting did increase IL-10 secretion. The therapeutic effects of PRGF were further investigated on nerve tissue regeneration by using a sciatic nerve transection model in rats. As was the case in the *in vitro* assays, the pro-inflammatory cytokines TNF-α, IL-1β and IL-6 were reduced in the PRGF group compared to the saline control group. In fact, treatment with the platelet preparation decreased M1-type macrophages, thus suggesting that its administration may modulate the inflammatory microenvironment to promote sciatic nerve regeneration via macrophage polarization among others.

### Murine macrophages

Fifteen investigations were included in this section. In most of them (12 out of 15), the results were that treatment with platelet derivatives modulated the polarization of macrophages towards the M2 phenotype. Only one article (75) reported a stimulation towards the pro-inflammatory phenotype. Nishio et al. (65) described both situations, depending on the type of PRP used. No modulation of macrophage polarization was reported by Hudgens et al. (59). The studies covered different fields of application and responses were evaluated both *in vitro* and *in vivo*.

### In vitro

Five studies explored the *in vitro* response of murine macrophages to different platelet derivatives (54, 59, 61, 64, 75). Both Cao et al. (54) and Kargarpour et al. (61) focused on bone regeneration, and although the treatments and the approach were different, their outcomes supported the same conclusion that platelet derivatives possessed anti-inflammatory activity by promoting macrophage M2 polarization. Cao et al. (54) evaluated the immunomodulatory role of PRP in combination with methacrylated gelatin (GelMA) and methacrylated alginate (AlgMA) (GA) hydrogel alone or together with Laponite nanoparticles. The authors concluded that M1 and M2-type genes were decreased and increased, respectively, for both PRP-containing hydrogels. Different fractions of liquid PRF were studied by Kargarpour et al. (61): platelet poor plasma (PPP), the buffy coat (BC or C-PRF), the remaining red clot (RC) and albumin gels (Alb-gel) from heating PPP. The results of their research indicated that lysates of both BC and PPP inhibited the inflammatory response of macrophages exposed to different TLR agonists, as evidenced by the significant decrease of IL-6 and COX-2. This was further confirmed with a reduction in p65 phosphorylation and NFκB nuclear translocation. Both types of lysates repolarized the M1-like macrophages as deduced from the increased expression of M2 phenotype markers, ARG1 and YM1. Not only did these fractions exert a potent anti-inflammatory effect but they also inhibited osteoclastogenesis, finding that has also been reported by other authors (55) as already mentioned above.

The study performed by Hudgens et al. (59) compared the effect of PPP and PRP on macrophage polarization in tendon disorders. In contrast to most studies, they used resident macrophages, specifically rat resident peritoneal macrophages. They concluded that PRP had no obvious effect on modulating macrophage polarization. However, as they suggested, multiple PRP doses should have been evaluated as well as changes in protein expression beyond the gene response.

In the context of tissue regeneration, Nasirzade et al. (64) studied the effect of PRF lysates, produced by freeze-thawing the membranes, and the PRF conditioned medium, that is, its secretome, on macrophages exposed to saliva and LPS. Their findings were in line with those already discussed from Kargarpour et al. (61) (authors from the same working group). Briefly, PRF possessed anti-inflammatory activity and shifted the macrophage polarization from M1 toward M2 phenotype. Further, NF-κB p65 signalling activation was strongly reduced by PRF lysates. The authors reported that the transition between M1 and M2 was partially mediated via activation of TGF-β signalling. They also described that PRF could modulate the expression of lipoxygenases (ALOX5, ALOX12 and ALOX15) thus supporting the production of pro-resolving lipid mediators. Still in the field of wound healing, Ulivi et al. (75) showed that PL supports macrophages in a proinflammatory state, thus enhancing the key initial inflammatory response to the injury. However, it should be noted that the outcome described in this article is not a direct but a paracrine effect of the PL. That is, the collected conditioned media from MSCs exposed to PL were used to treat bone marrow-derived macrophages and to assay its effect on their phenotype.

### *In vitro* and *in vivo*

In the field of bone healing, the M2 polarized macrophages are critical in the osteogenic microenvironment for effective bone regeneration (91). In this sense, Li et al. (62) developed hybrid hydrogels composed of injectable platelet-rich fibrin (i-PRF) and polycaprolactone/hydroxyapatite composite nanofibers by using enriched polydopamine (PDA) as linker (PnP-iPRF). To simulate the biodegradation process *in vivo*, i-PRF-containing hydrogels were treated with plasmin. RAW 264.7 macrophages were then incubated with those degradation products. The authors reported that the immunomodulatory activity of the PnP-iPRF could be attributed to the PDA component, as this hybrid hydrogel effectively induced M2 macrophage activation in a sustained manner. Cytokines secreted by these PnP-iPRF-treated macrophages also promoted the osteoblastic differentiation of BMSCs. PnP nanofibers were considered to be responsible for enhancing this osteogenic activity of i-PRF hydrogels. However, i-PRF hydrogel produced a moderate and unsustainable induction towards the M2 phenotype. The authors created a critical cranial defect in rats to further investigate the osteogenic effect of this hybrid hydrogel *in vivo*. The results were in line with those obtained in the *in vitro* assays. PnP-iPRF hydrogels could effectively induce M2 macrophage phenotype at 4 weeks post-surgery, as deduced from expression of CD206 and iNOS, markers of M2 and M1 respectively. Therefore, in this study, the added value was provided by the PnP rather than by the i-PRF.

Leukocyte rich PRP was select by Jiang et al. (60) for incorporation into the GelMA hydrogel on macrophages and to study its resulting influence on osteochondral regeneration. The addition of 20% PRP-GelMA significantly reduced the *in vitro* expression of several M1 markers (IL-1β, IL-6, iNOS, CCR7) in the LPS-treated macrophage culture when compared with pure GelMA or control. In this regard, M2 markers (Arg-1, IL-1ra, IL-10 and CD206) exhibited higher expression in PRP-GelMA than in the other groups. The authors indicated that the PRP-GelMA hydrogel not only inhibited the transition from M0 to M1 but also promoted M2 polarization. This hydrogel combination was further investigated in a rabbit model of osteochondral defect and showed early onset (12 weeks) and the persistence (18 weeks) of the M2c macrophage subset, and the reduction of M1 phenotype, thus suggesting that osteochondral regeneration was mediated by M2 polarization. Although not directly comparable, as the composition of PRP and hydrogel were different, the addition of the platelet derivative in this study did lead to an improvement in the performance of the hydrogel in contrast to that reported by Li et al. (62).

Macrophage infiltration and polarization have been increasingly associated with the degree of intervertebral disc degeneration (IDD) (92). Therefore, Qian et al. (70) addressed the underlying mechanisms involved in IDD pathology. They compared the effect of PRP with that of PRP-derived exosomes. The authors showed that both PRP and PRP-derived exosomes inhibited the polarization of M1-type macrophages through the inhibition of genes associated with this pro-inflammatory phenotype. This occurred via suppressing NF-κB and MAPK signalling by targeting TRAF6. The inhibitory ability was more pronounced in the case of PRP-derived exosomes. On the other hand, PRP-derived exosomes promoted M2 macrophage polarization via the STAT6 signaling pathway. The strongest inhibitory effect was exerted with respect to IL-1β, which led the authors to explore the effect of PRP-derived exosomes on NLRP3 inflammasome in macrophages. The NLRP3 inflammasome is a multiprotein complex critical in the innate immune system that assembles in response to pathogens and other stressors leading to the activation of caspase-1, the secretion of proinflammatory cytokines (Il-1β, IL-18) and the induction of pyroptosis (93–95). The authors reported the inhibition of NLRP3 inflammasome activation by PRP-derived exosomes. They also used rat models of IDD to address the issue *in vivo*. As it was reported *in vitro*, the expression of inflammasome-related proteins was also reduced in the PRP-derived exosomes group *in vivo*. The results of this study differ somewhat from those obtained by Graca et al. (57) where the extracellular vesicles did not clearly polarize towards the M2 phenotype. However, the comparison between these two studies is challenging, since the origin of the vesicles, PRP and macrophages is different, as well as the platelet derivative’s composition.

Several authors (72, 79, 80) have carried out studies in terms of wound healing and despite their different approaches, all results consistently showed that platelet derivatives stimulated polarization towards the M2 phenotype. Tang et al. (72) developed a thermosensitive injectable hydrogel known as the thermosensitive decellularized adipose tissue/platelet-rich plasma interpenetrating polymer network (t-DPI) hydrogel based on decellularized adipose tissue (DAT) and temperature-controlled platelet-rich plasma (t-PRP). All the treatments (t-PRP, DAT hydrogel and t-DPI hydrogel) promoted M2 macrophage polarization *in vitro* compared to the control. However, the t-DPI hydrogel group was the treatment that induced the highest polarization. Same results were obtained *in vivo* in a nude mouse model. The authors concluded that the biologically active ingredients present in the t-PRP and DAT contributed to the therapeutic effect of t-DPI hydrogel via M2 macrophage polarization, among others. On the other hand, Zhang et al. (79) concluded that i-PRF had greater anti-inflammatory response than whole blood (WB). In fact, i-PRF reduced the expression of IL-6, TNF-α, and INOS2 in LPS-treated murine-derived macrophage compared with WB. Moreover, p65 phosphorylation and TLR4 expression was also decreased after treatment with this platelet concentrate. The inhibition of NFκB signalling was consistent with that reported by other authors already included in this review (61, 64, 70, 71), that also showed the ability of the different platelet derivatives to stimulate the M2 phenotype. In this study, i-PRF also increased the M2-polarized macrophage phenotype-associated cytokines. Results also showed an anti-inflammatory response *in vivo*. i-PRF reduced the amount of local innate immune cells in a rat muscle defect model. Finally, Zhao et al. (80) evaluated PRP as an additive to develop an alginate-gelatin (AG) composite hydrogel bioink. Different concentrations of PRP were incorporated. Macrophages were seeded on the 3D bioprinted double-layered skin substitutes under inflammatory conditions (LPS). The addition of 5% PRP (AG-5P) led to greater decrease and increase of iNOS and Arg-1, respectively, than the control and AG groups. The same results were obtained in the *in-situ* 3D bioprinting repair of a rat dorsal full-thickness wound model. In addition, the AG-5P reduced the inflammatory response and macrophage infiltration after transplantation, as suggested by the lowest level of CD3, CD68 and MPO that were detected compared with control and AG group. Therefore, the authors concluded that the PRP incorporation regulated the immune response and tissue regeneration by promoting the macrophage polarization towards an M2 phenotype.

### In vivo

Four articles were included in this section. Despite the fact that the experimental design and the type and composition of PRP were different, 3 out of 4 studies concluded that platelet derivatives stimulated the anti-inflammatory phenotype. In the case of the study of Nishio et al. (65) polarization depended on the type of PRP, as LP-PRP induced the M2 phenotype, while LR-PRP induced the pro-inflammatory M1 phenotype.

In line with the studies of hybrid hydrogels, Qian et al. (69) reported the combination of PRP and lyophilized platelet-rich fibrin (Ly-PRF) with alginate-hyaluronic acid hydrogel as a novel approach for the treatment of myocardial infarction. They concluded that in this cardiovascular condition, Ly-PRF improved the immunological response by reducing the M1 macrophages and by stimulating their polarization towards the M2 phenotype.

Park et al. (68) evaluated the effect of PRP on an acute UVB-induced skin photodamage model in rats. They reported that the addition of PRP modulated the immune response depending on the stage of wound healing. That is, PRP enhanced the inflammatory or the repair response 7 days or 28 days after treatment, respectively, thus, finally reducing skin tissue inflammation. This regulation of macrophage polarization was performed via the activin receptor-follistatin system.

Two studies (65, 78) addressed tendon healing. As already mentioned, Nishio et al. (65) evaluated the influence of the leukocyte inclusion in the PRP on macrophage recruitment and polarization. They created full-thickness defects in the central third of patellar tendons in mice. As already described in the findings of Park et al. (68), macrophage modulation was dependent on the stage of tendon healing. They demonstrated that both types of PRP enhanced the tendon healing and promoted the recruitment of macrophages to injured tissue. However, leukocytes did influence the effect that PRP has on the balance between M1 and M2. In fact, LR-PRP preferentially stimulated the activity of the M1 phenotype, whereas LP-PRP did so with M2 macrophages. Nevertheless, the authors also noted that the effect of the PRP groups could be due to a foreign body reaction as the control group was not well-designed. On the other hand, Yu et al. (78) evaluated the effect of PRP releasate (released from platelets after PRP was activated) on the early stages of tendon healing. A rat model of Achilles tendon injury was used. In contrast to Nishio et al. (65), saline solution was used as a control. They reported lower levels of CD68^+^ (ED1^+^) macrophages, which have been suggested to stimulate tendon catabolism, in the samples treated with PRP as compared with the control group in the early healing stage (day 5 post injury), thus promoting tissue recovery.

### Ovine macrophages

Wessely-Szponder et al. (76) used ovine monocyte-derived macrophage cultures to assess the influence of autologous ovine PRP and rabbit antimicrobial peptide extracts. Macrophages were previously stimulated with LPS or dexamethasone. The proinflammatory cytokines superoxide and nitric oxide (NO) were increased in macrophage cultures after PRP treatment both alone and in combination with LPS, although the addition of the latter exacerbated this response. The use of dexamethasone reduced the proinflammatory response to PRP. Therefore, the authors concluded that PRP treatment induced a pro-inflammatory rather than repair phenotype rather than a repair one. Although this response might enhance antimicrobial activity, they suggested that the application of this platelet derivative should be restricted in cases of severe inflammation.

## Discussion and future perspectives

The therapeutic potential of promoting platelet-macrophage interactions is still a matter of debate. Macrophages are tissue-resident or infiltrated immune cells critical for innate immunity, normal tissue development, homeostasis, and tissue repair (5). The macrophage population in adults was originally thought to derive solely from circulating monocytes originating in the bone marrow. However, accumulating evidence has redefined this paradigm. Tissue-resident macrophages (TRMs) derive from yolk sac and fetal liver progenitors, and bone marrow-derived monocytes (12–14, 96). Therefore, ontogenetically distinct macrophages coexist in human adult tissues leading to a cellular mosaic that is dynamically modulated throughout life (9). Infiltrating monocyte-derived macrophages are functionally and phenotypically distinct from TRMs (34). In fact, when the former are recruited in a pathological process, they encounter a much more distinct milieu than TRMs (34). Under both normal and pathological conditions, the contribution of these distinct sources of macrophages varies in a tissue-specific manner (96). Macrophage function is a sum of their ontogeny, tissue-specific environmental signals, and the type of injuries to which they are exposed. All together contributes to shaping macrophage transcriptional regulation and functional specialization (5, 96, 97). Despite this macrophage heterogeneity, the cellular and molecular responses have been mainly studied on the monocyte-derived population, ignoring the peculiarities of tissue resident macrophages and their ontogeny. Therefore, it seems critical to identify reliable markers to distinguish the different subsets of TRMs and the infiltrating monocyte-derived macrophages to more precisely address the research of macrophages-mediated response and their multiple interactions (34). Most of the research available for this review studied the effects of the different platelet derivatives on the monocyte-derived macrophages, and those using resident macrophages (59, 61) also did not specify their ontogeny, thus, missing the final pieces of the puzzle.

Beyond the aforementioned ontogeny, comparison of results was also very challenging for several reasons. Firstly, the great variability of protocols for obtaining platelet derivatives leads to products with different compositions; that information was also missing in many studies, thus further confusing the interpretation of the results. Protocols for obtaining M1 and M2 phenotypes, including inducer molecules and treatment times, also varied widely among studies which could influence the secretome released by polarized macrophages thus affecting the outcome of the immune challenge. The origin of species might be another intrinsic factor that can modulate the biological response of macrophages. Two different species have been mainly studied in this review: human and murine. Mice are the experimental system of choice for most immunological studies. In fact, they have contributed extensively to the understanding of the human immune system, with the genomes of both species being highly conserved. However, there are also significant differences in their immune systems in terms of development, activation and response. Therefore, while these mouse models will continue to be used to provide new knowledge on the subject, there is also an increasing need for a cross-species approach to determine the potential limitations in translating data to humans (98). On the other hand, among rodent models, both rats and mice of different strains are used. It has already been reported that macrophages from different strains differ in the type of immune response (99, 100). C57BL/6 and Balb/c mice are two of the most commonly used strains, as also reflected in the current review, that differ in their immune responses. The former is more reactive, giving prototypical Th1-biased immune responses whereas Balb/c mice give Th2-biased immune responses (101, 102). This further complicates the interpretation of the data. The type of cells used to generate macrophages could also add a further level of difficulty.

Despite the challenges of comparing data, the studies covered in this review mostly showed that platelet derivatives promoted macrophage polarization towards the pro-repair M2 phenotype. There was no apparent difference in immune response among the different types of platelet derivatives (PRP, PL and PRF). Nor did the composition of various preparations showed clear differences in terms of leukocyte inclusion. In other words, in the light of the studies included in this review, the inclusion of white cells did not induce a M1 phenotype to a greater extent. However, assumptions on this issue should be assessed with caution, as many of the studies did not specify whether they included leukocytes or not. Furthermore, in studies that did provide details, the conditions under which the platelet-derived products were obtained varied greatly, further complicating the comparison between them. Ideally, every study should have included the same platelet derivative with and without leukocytes for the results to be reliable. However, this only occurred in a total of 3 articles (65, 67, 74), of which only Nishio et al. (65) found different outcomes between the two products, as leukocyte-rich PRP mainly enhanced the effects of M1 macrophages compared to leukocyte-poor product that stimulated M2 phenotype. Furthermore, the white cell content reported by Papait et al. (67) was practically nil in both preparations. On the other hand, the M1/M2 dichotomy is an over-simplification, and therefore might not serve as a potency testing to truly assess the effect of leukocytes on platelet derivatives *in vivo*, in which a “continuum” of activation states exists.

Several studies (54, 60, 62, 69, 72, 80) have also focused on developing hybrid hydrogels that combine platelet derivatives with biomaterials from different origins, with the aim of complementing and optimising their immunoregulatory functions. In all cases, the results of the combination were similar, that is, stimulation towards the M2 phenotype. All the authors considered the incorporation of the platelet derivatives as an added value in the composite hydrogel development to improve the immunoresponse, except for Li et al. who attributed the immunomodulatory activity to the PDA component. Nevertheless, only two of the articles (62, 72) included the platelet derivative as a control group in their study, so that the relative contribution of the blood derivative to the composite could be deduced.

As already mentioned, the widespread use of M1/M2 nomenclature is an over-simplified description of macrophage heterogeneity. This model was introduced to describe the two different macrophage responses, reflecting the T-helper cell nomenclature (103). However, macrophage polarization is a complex dynamic process with this M1-M2 binary model representing only the extremes of the spectrum (104). Nowadays the identification of other subsets of unconventional macrophages such as tumor associated macrophages (TAMs), macrophages expressing T cell receptors (TCR) and CD169 has added complexity to the issue (105, 106). In fact, a small group of macrophage biologists met at the International Congress of Immunology in Milan in August 2013 and proposed a common framework for macrophage activation nomenclature (107) in an attempt to resolve areas of confusion and to establish an initial set of experimental guidelines. Recommendations encompassed the following: a reproducible *in vitro* experimental standard, minimal reporting standards, definition of the activator, avoidance of certain terms and inclusion of markers of activation. However, even though most of the articles included in this review provided fairly complete information on *in vitro* assays (**Table 3**), some studies lacked details on cell density, culture medium, etc. The focus on whether a certain treatment stimulates the M1 or M2 phenotype does not clearly reflect the biological complexity, given that M1-like macrophages may participate in the tissue repair process, depending on the circumstances in which they occur and the length of time that they remain. Thus, it may be more informative to focus on the ratio or balance between the two phenotypes or on the ability of a given treatment to shift polarization towards the M2 phenotype.

Macrophage polarization is a tightly controlled process that is regulated by multiple signalling cascades and transcription factors to achieve an optimum and dynamic balance between macrophage subpopulations (24, 108). In this review, several mechanisms have been proposed to be involved in the immunoregulatory role of platelet derivatives in controlling macrophage polarization. Both human and murine macrophages share signalling pathways. For the former, polarization towards M2 phenotype was reported via PI3K/AKT signalling pathway activation (63), or by reducing the expression of NF-κB, or by increasing STAT3 and STAT6 and SMAD2 and SMAD4 expression (71), thus involving TLRs/NF-κB, JAK-STAT, and TGF−β signalling pathways, respectively. Regarding murine macrophages, the main transcription factor involved in the inflammatory response, namely NF-κB, was also reported its signalling reduction in several studies (61, 64, 70, 79), thus promoting the polarization of M2-like macrophages. Besides the TLR/NF-κB signalling pathway, Qian et al. (70) also described the involvement of the JNK and JAK-STAT signalling pathways in M1 and M2 phenotypes, respectively. In fact, they showed that PRP-derived exosomes inhibited M1 macrophage polarization by inactivating NF-κB and MAPK pathways and targeting TRAF6 and promoted the polarization toward M2-type macrophage via the STAT6 signalling pathway. Nasirzade et al. (64) also showed that the transition from M1 to M2 was partially due to an activation of TGF-β. This growth factor-related signalling pathway was also involved in the regulatory role of PRP through affecting activin activity (68).

In summary, macrophages are extremely plastic immune cells involved in tissue homeostasis and pathological conditions. As a consequence, they represent relevant therapeutic targets. To this end, several approaches have been proposed, including pharmacological interventions, transplantation of specific subsets of macrophages, epigenetic modifications, genetic engineering or depletion of NLRP3 inflammasome (109, 110). In this sense, platelet derivatives that have been successfully applied in many medical fields for decades, might be another option to consider. However, the heterogeneity of these biological therapies, due to differences in preparation protocols, cell content or platelet activation status, and inconsistences in nomenclature, has contributed to different clinical outcomes (111). In addition, most of the published studies do not provide all the information necessary for protocol reproducibility. In the same vein, the standardization and reproducibility of macrophage isolation, activation and polarization should be a requirement for the development of new therapies. Furthermore, understanding the differences between human macrophages and those derived from animal models seems essential for the effective design of new therapeutic strategies (105). Similarly, monocyte-derived macrophages do not fully represent what occurs in humans; thus, understanding the complex relationships between tissue-resident macrophages and infiltrating monocyte-derived macrophages and their relative contribution to homeostasis and disease might provide critical insights to platelet derivatives’ immunomodulatory response. Reprograming macrophage phenotypes is considered to be a promising strategy for designing novel therapies rather than those focusing on eliminating these immune cells (33). In this regard, and on the basis of the results derived from this review, platelet derivatives could play an important role in inducing a dynamic M1/M2 balance and promoting a timely M1-M2 shift. Therefore, despite the issues that remain to be resolved, combination of macrophages and platelet derivatives provides relevant information on the function and mechanisms of the immune response.

## Author contributions

EA: Conceptualization, Supervision, Writing – review & editing. MT: Data curation, Formal analysis, Investigation, Methodology, Software, Writing – original draft, Writing – review & editing. MA: Conceptualization, Supervision, Writing – review & editing.
